# Long-Term Cardiac Safety and Survival Outcomes of Neoadjuvant Pegylated Liposomal Doxorubicin in Elderly Patients or Prone to Cardiotoxicity and Triple Negative Breast Cancer. Final Results of the Multicentre Phase II CAPRICE Study

**DOI:** 10.3389/fonc.2021.645026

**Published:** 2021-07-09

**Authors:** Miguel J. Gil-Gil, Meritxell Bellet, Milana Bergamino, Serafín Morales, Agustí Barnadas, Luís Manso, Cristina Saura, Adela Fernández-Ortega, Elena Garcia-Martinez, Noelia Martinez-Jañez, Mireia Melé, Patricia Villagrasa, Pamela Celiz, X. Perez Martin, Eva Ciruelos, Sonia Pernas

**Affiliations:** ^1^ Department of Medical Oncology, Institut Català d’Oncologia, IDIBELL, L’Hospitalet, Spain; ^2^ Department of Medical Oncology, Hospital Vall d’Hebron, Barcelona, Spain; ^3^ Department of Medical Oncology, Hospital Arnau de Vilanova, Lleida, Spain; ^4^ Department of Medical Oncology, Hospital de Sant Pau, Barcelona, Spain; ^5^ Department of Medical Oncology, Hospital, 12 de Octubre, Madrid, Spain; ^6^ Department of Medical Oncology Hospital, JM Morales Messeguer, Murcia, Spain; ^7^ Department of Medical Oncology, Hospital Ramón y Cajal, Madrid, Spain; ^8^ Department of Medical Oncology, Hospital Sant Joan, Reus, Spain; ^9^ SOLTI Breast Cancer Research Group, Barcelona, Spain; ^10^ Clinical Research Unit, Institut Català d’Oncologia, L’Hospitalet, Spain

**Keywords:** pegylated liposomal doxorubicin, elderly, neoadjuvant chemotherapy, triple negative breast cancer, long-term results, cardiotoxicity, survival, phase II study

## Abstract

**Background:**

The CAPRICE trial was designed to specifically evaluate neoadjuvant pegylated liposomal doxorubicin (PLD) in elderly patients or in those with other cardiovascular risk factors in whom conventional doxorubicin was contraindicated. The primary analysis of the study showed a pathological complete response (pCR) of 32% and no significant decreases in LVEF during chemotherapy. Here, we report important secondary study objectives: 5-year cardiac safety, disease-free survival (DFS), overall survival (OS) and breast cancer specific survival (BCSS).

**Methods:**

In this multicentre, single-arm, phase II trial, elderly patients or those prone to cardiotoxicity and high risk stage II-IIIB breast cancer received PLD (35 mg/m^2^) plus cyclophosphamide (600 mg/m^2^) every 4 weeks for 4 cycles, followed by paclitaxel for 12 weeks as neoadjuvant chemotherapy (NAC). Left ventricular ejection fraction (LVEF) monitorization, electrocardiograms and cardiac questionnaires were performed at baseline, during treatment and at 9, 16, 28 and 40 weeks thereafter. The primary endpoint was pCR and 5-year cardiac safety, DFS, BCSS and OS were also analyzed.

**Results:**

Between Oct 2007, and Jun 2010, 50 eligible patients were included. Median age was 73 (35-84) years, 84% were older than 65; 64% of patients suffered from hypertension, and 10% had prior cardiac disease. Most of tumors (88%) were triple negative. No significant decreases in LVEF were observed. The mean baseline LVEF was 66.6% (52-86) and after a median follow-up of 5 years, mean LVEF was 66 (54.5-73). For intention to treat population, 5-year DFS was 50% (95% CI 40.2-68.1) and 5-year OS was 56% (95%CI 41.2-68.4). There were 8 non-cancer related deaths, achieving a 5 years BCSS of 67.74% (CI 95%:54.31%- 81.18%).

**Conclusion:**

At 5-year follow-up, this PLD-based NAC regimen continued to be cardiac-safe and effective in a population of very high-risk breast cancer patients. This scheme should be considered as an option in elderly patients or in those with other risks of developing cardiotoxicity.

**Trial Registration Number:**

ClinicalTrials.gov reference NCT00563953.

## Introduction

Several randomized trials and a meta-analysis confirmed that chemotherapy regimens that include anthracyclines are more effective than those without them (1). Despite their high antitumor activity profile, their use in clinical practice is limited due to potential cardiotoxicity (2). Conventional doxorubicin (DOX) can induce myofibrillar damage with a decrease in Left Ventricular Ejection Fraction (LVEF), leading to irreversible congestive heart failure (CHF) (3). The mechanisms of cardiotoxicity of DOX are mainly caused by the pro-apoptotic effect of the topoisomerase II inhibition in the cardiomyocytes resulting in loss of functional myocytes and irreversible heart injury (4). The risk of developing chronic anthracycline-induced cardiotoxicity is strongly related to several factors. The main risk factor is the cumulative dose of this agent, but one prospective study revealed that only 240 mg/m^2^ of accumulated DOX caused grade 1 cardiotoxicity in 17% patients and grade 2 in 6.6% (5). Some retrospective studies showed that in addition to the accumulated dose, the risk of CHF correlate with the age of the patient and with the scheme of administration (6). Continuous infusion schemes have been reported to be less cardiotoxic compared with rapid infusion of DOX (7). Other independent risk factors for developing cardiomyopathy are pre-existing cardiac disease and history of hypertension.

Pegylated liposomal doxorubicin (PLD), trademark Caelyx/Doxil^®^, is an encapsulated anthracycline that was designed to reduce toxicity of DOX while preserving its antitumor efficacy by altering its tissue distribution and pharmacokinetics. It is associated with a significantly reduced incidence of cardiotoxicity (8, 9). There are at least two known factors that contribute to the less cardiotoxic profile of the liposome-encapsulated anthracyclines. First, this formulation reduces the amount of drug reaching the cardiac cells and secondly, it enables a slow release of DOX that avoid peak plasma concentrations. PLD has longer half-life (50-80 hours) than non-pegylated liposomal DOX (10-16 hours) and conventional DOX (10 minutes) (10, 11). PLD has demonstrated to have equivalent efficacy but significantly less cardiotoxicity than conventional DOX in a phase III non-inferiority trial in patients with metastatic breast cancer (12). In addition, a meta-analysis including data from nine randomized trials of different tumors, compared the efficacy and safety profile of liposomal formulations versus conventional anthracyclines. In this meta-analysis, liposomal DOX and PLD demonstrated favorable toxicity profiles with better cardiac safety and less myelosuppression, alopecia, nausea, and vomiting compared to conventional anthracyclines, without compromising effectiveness (13).

Given the projected substantial increase of the elderly population over the following years, it is necessary to develop new chemotherapy regimens with equivalent efficacy, and better tolerability, for this vulnerable subset of patients. We conducted a phase II, single-arm, multicenter clinical trial to evaluate the efficacy and safety of a NAC based on PLD plus cyclophosphamide followed by paclitaxel in patients with breast cancer and at high risk of developing cardiotoxicity: the SOLTI-CAPRICE study (NCT00563953). The results of complete pathological response (pCR), breast conserving surgery (BCS) and acute toxicity have already been published (14). This is an update on the cardiotoxicity and survival outcomes at five years of follow-up which were secondary objectives of the study.

## Patients and Methods

### Study Design

Phase II, Open-Label, Multicentre Clinical Trial aimed to evaluate PLD plus Cyclophosphamide followed by paclitaxel as NAC for breast cancer patients with risk of developing cardiotoxicity. The protocol was designed and approved by the ethics committee at each participating center in 2007.

### Eligibility Criteria

Histologically confirmed stage II-IIIB invasive breast cancer, estrogenic receptor (ER) below 50% and at least one of the following cardiotoxicity risk conditions: > 65 years old, pre-existing heart disease, LVEF <55%, clinical history of hypertension requiring pharmacological treatment, previous mediastinal irradiation or previous treatment with anthracyclines. Full eligibility criteria are published online (**Table S1**).

### Treatment Plan and Evaluations

**Figure 1:**
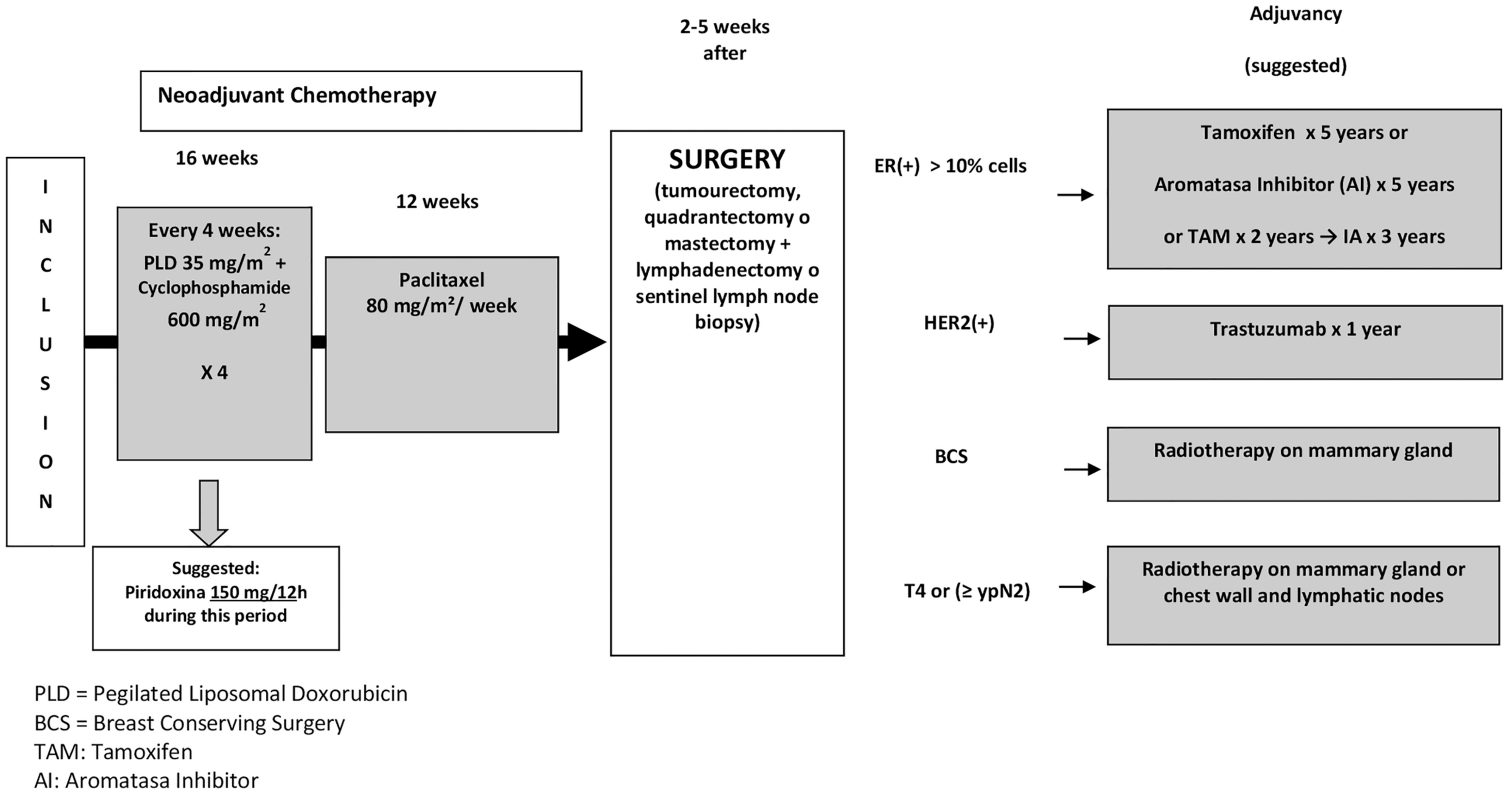
Scheme of the Study. PLD, Pegylated liposomal doxorubicin; ER, Estrogen receptors; HER2, Human epidermal growth factor receptor 2; BCS, Breast conservative surgery; TAM, Tamoxifen; N, Node; IA, Aromatase inhibitors.

NAC regimen was based on 4 cycles of PLD 35 mg/m^2^ plus cyclophosphamide 600 mg/m^2^ on day 1 every 4 weeks followed by paclitaxel 80 mg/m^2^/week for 12 weeks (**Figure 1**). Dose reduction criteria is summarized in **Table S2**.

Physical examination, LVEF monitorization, electrocardiogram (ECG) and cardiac questionnaire were performed at the beginning of the study and during weeks 9, 16, 28; then every 3 months for 2 years and then every 6 months until the 5th year. Cell blood count, chemistry profile, physical examinations, and toxicity assessments were performed every 4 weeks during treatment. Toxicities were graded using the Common Terminology Criteria for Adverse Events (CTCAE) 3.0 common toxicity criteria.

The type of surgery indicated by the surgeon prior to NAC initiation was recorded, and surgery treatment was performed between 2-5 weeks after the last infusion of NAC

### Response Criteria

Surgical specimens were evaluated for pathological tumour response according to the NSABP guidelines (15). pCR was defined as no evidence of residual invasive cancer (ypT0/is) in the breast specimen while breast and axilla pCR was defined as the absence of invasive disease in the breast and axillary nodes (ypT0/Tis ypN0). Radiological response was evaluated according to WHO criteria.

### Statistical Analysis

The primary end point of the study was the pCR rate in breast. To determine the sample size, the expected proportion of complete pathological responses (pCR) was assumed to be higher than 18%. It was also established that the investigational regimen would be of no further interest if the true pCR rate was < 8% (H_0_). The alternate hypothesis (H_A_) assumed that a true pCR rate ≥ 18% would be of considerable interest in breast cancer patients with high risk of developing cardiotoxicity. The hypothesis test was resolved with a risk α = 0.05, using a conformance test of a proportion (π = 0.08) versus unilateral alternative (ß = 0.08) based on the Normal approximation. They provided the lower limit of the confidence interval (unilateral) of 95% for the proportion of pCR. Assuming a 10% drop out rate, the sample needed was calculated in 66 patients.

Secondary end points of the study were: Cardiac safety measured by decrease in LVEF, overall response rate (ORR) after PLD plus Cyclophosphamide, ORR at the end of the NAC, BCS rate, pCR in breast and axilla, and 5-year relapse free survival (RFS) and overall survival (OS). More information can be found in **Table S3**.

## Results

Between October 2007 and June 2010, 51 patients signed the informed consent. The sample size was lower than initially foreseen due to slow recruitment. One patient was excluded before treatment initiation because of metastatic disease identified at baseline. Therefore, 50 patients were included in the intent-to-treat (ITT) analysis.

Patient and tumors characteristics are summarized in **Table 1**. It is noteworthy that 84% of the patients were older than 65 years old and that 88% of the tumors were triple negative. At baseline only 13 patients (26%) were candidates for BCS. Twenty-six (52%) breast cancer cases were stage III, including 7 (14%) inflammatory breast carcinomas and 14 (28%) T4b-c.

**Table 1:** Patient and tumor baseline characteristics.

		n	%
Patients in *ITT*		50	100
* Age:*	<60 years	6	12
	60-64 years	2	4
	65-69 years	11	22
	70-74 years	11	22
	75-79 years	14	28
	>79 years	6	12
**ECOG PS**	0	43	86
	1	7	14
**Menopausal status**			
	Pre	4	8
	Post	46	92
Mean **tumor size** in mm (range)		33.7 (5-123)	
**Histological Grade**	G1	4	8
	G2	10	20
	G3	36	72
**ER–status**	0%	42	84
	1-9%	2	4
	>10%	6	12
**HER2/neu**	negative	49	98
	positive	1	2
**Disease stage**	II	24	48
	III	26	52
**T clinical**	T2-T3	29	54
	T4 a-c	14	28
	T4d	7	14
**N clinical**	N0	23	46
	N1	14	28
	N2	11	22
	N3	2	4
**Cardiotoxicity risk factors**	age > 65	42	84
	hypertension	32	64
	prior cardiac disease	6	12
	lipid metabolic disorder	23	46
	diabetes	12	24
	*Brain Transient Ischemic Attack*	2	4
**Type of surgery** indicated in baseline			
	Mastectomy	37	74
	BCS	13	26

ITT, intention to treat. ECOG PS, Easter Cooperative Oncology Group Perfomance Status. BCS, breast conserving surgery.

**Figure 2:**
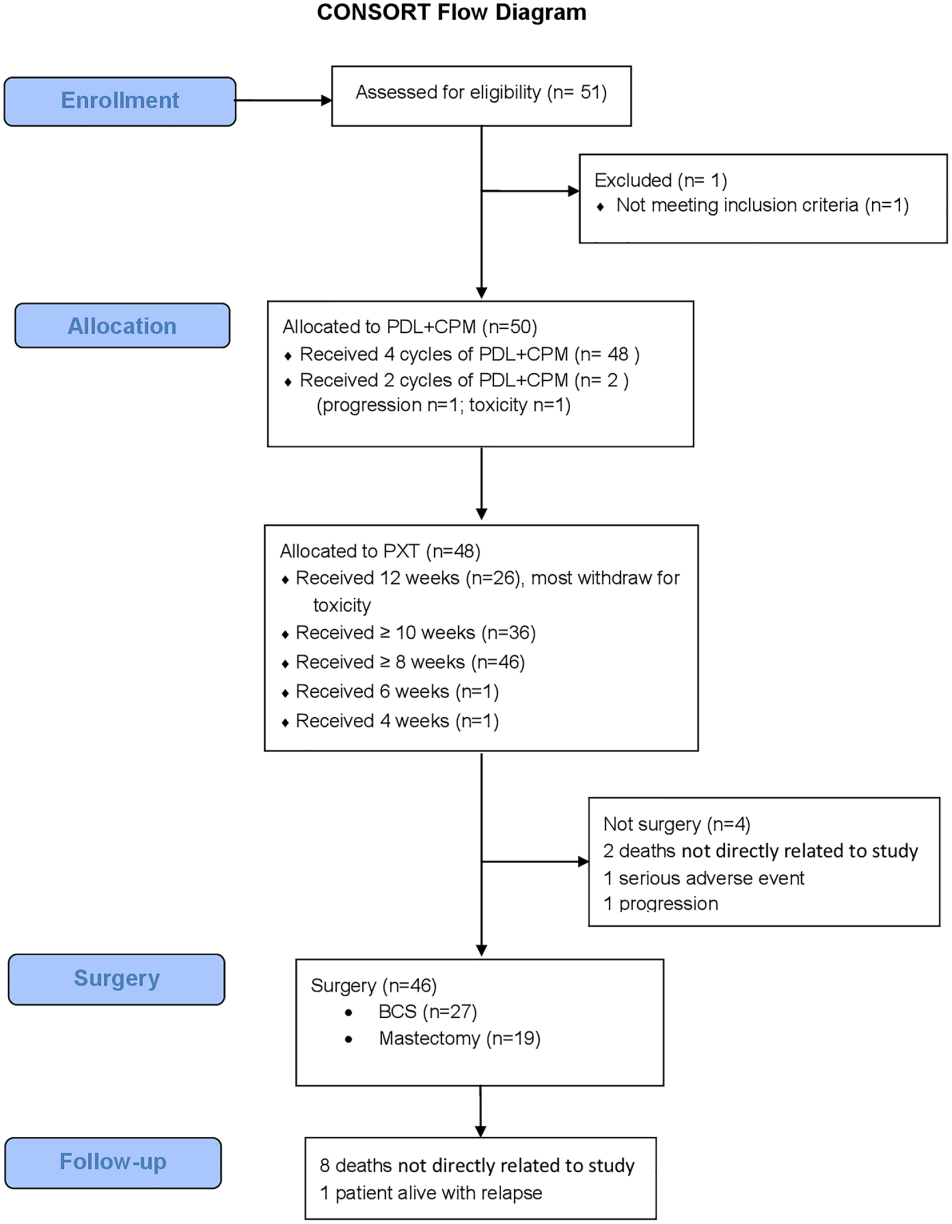
Consort flow diagram. n, number; PLD, Pegylated liposomal doxorubicin; CPM, Cyclophosphamide; PTX, Paclitaxel; BCS, Breast Conservation Surgery.

Forty-eight patients (96%) completed the 4 cycles of PLD plus cyclophosphamide, in whom eight patients (16%) required delayed or reduced doses. Only 26 patients (52%) were able to complete the 12 weeks of paclitaxel (**Figure 2**).

Forty-six patients (92%) underwent surgery: BCS was performed in 27 (58.7%) and mastectomy in 19 cases (41.3%). Thirty-nine patients (84%) underwent axillary lymphadenectomy and seven sentinel lymph node biopsies.

In the ITT analysis, the pCR rate in breast was 32% (95% CI 19.5-46.7%). This result allows us to reject the pre-specified null hypothesis (pCR ≤ 8%) with *p* < 0.0001 and proven alternative hypothesis (pCR rate ≥ 18%). Among triple-negative tumours (n=44) pCR was 33.3%. Two out of seven (28.7%) inflammatory breast carcinoma achieved a pCR. pCR in breast and nodes was 24% for ITT patients (95% CI 12.1-35.8%) (**Table S4**).

**Figure 3:**
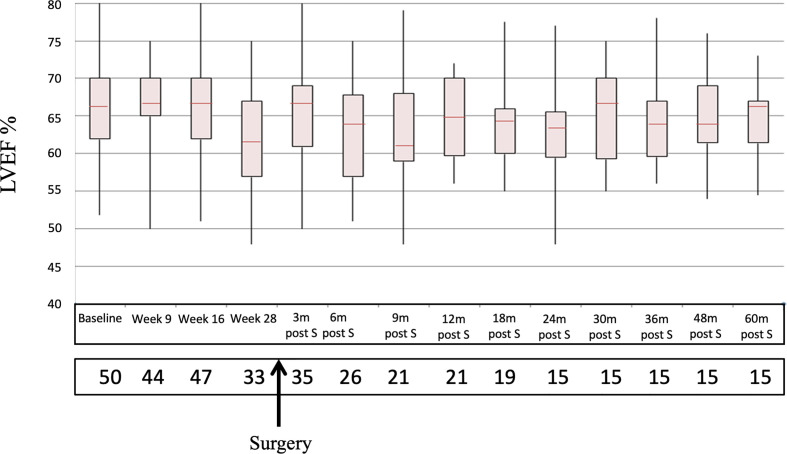
Sequential LVEF determinations since baseline to 60 m post-S. LVEF, Left Ventricular Ejection Fraction; M, months; post-S, post surgery.

No significant decreases in LVEF were observed. The mean baseline LVEF was 66.6 (52-86). After 16, 28, and 40 weeks LVEF was 66.7 (51-88), 62.2 (48-75), and 64.7 (50-74), respectively (**Figure 3**). Mean LVEF after 5 years follow-up was 66 (54.5-73). No relevant changes were observed in the ECG follow up either. A total of 406 adverse events (37 grades 3-4) were reported in 38 patients (**Table 2**). Four patients (8%) developed early cardiotoxicity (≥G3 in two cases): one sudden death in a 82-year-old patient three months after completion of NAC, one episode of atrial fibrillation and CHF during paclitaxel treatment, one case of palpitations and one case with a decrease in LVEF <15% versus prior value (toxicity grade 1).

**Table 2:** Most frequent toxicities.

Adverse Event	All grades		Grade 3-4	
	n	%	n	%
Fatigue	42	84	3	6
Alopecia	35	70	0	0
Neurology (sensitive)	31	62	3	6
Stomatitis	29	58	0	0
Nausea/Vomiting	25	50	1	2
Pain	21	42	0	0
Diarrhoea	17	34	2	4
Nail Changes	17	34	0	0
Anorexia	17	34	0	0
Skin reaction (rash)	16	32	2	4
Neutropenia	11	22	1	2
Hand-Food Syndrome	7	14	0	0
Oedema	7	14	0	0
Constipation	7	14	0	0
Fever	7	14	1	2
Haemolysis	6	12	1	2
Infection	6	12	1	2
Cardiac Event	5	10	3	6
Pulmonary	4	8	2	4
Neurology (other)	4	8	1	2
Anaemia	3	6	2	4
Hypersensitivity	3	6	1	2
Haemorrhage	3	6	2	4
Renal Failure	2	4	1	2

Overall, 24 patients died during the follow-up. Seventeen were cancer-related deaths and eight were 8 non-cancer related deaths (16%). Three out of these 8 non-cancer related deaths occur early in time, and all were in patients older than 80 years: a sudden death one month after surgery, a hemorrhagic stroke 30 days after completing paclitaxel, and a non-neutropenic pneumonia during paclitaxel course. An Independent Multidisciplinary Safety Committee evaluated the cause of death in the three cases and concluded that it was probably due to old age and not directly related to the study treatment. There were subsequently five non-cancer related deaths during the follow-up: a case of amyotrophic lateral sclerosis (ALS), an stroke, an intestinal ischemia, a heart failure due to valvular disease in a 85-year-old patient and an unknown-cause death in a 83-year-old woman after nine years of the study treatment end.

**Figure 4:**
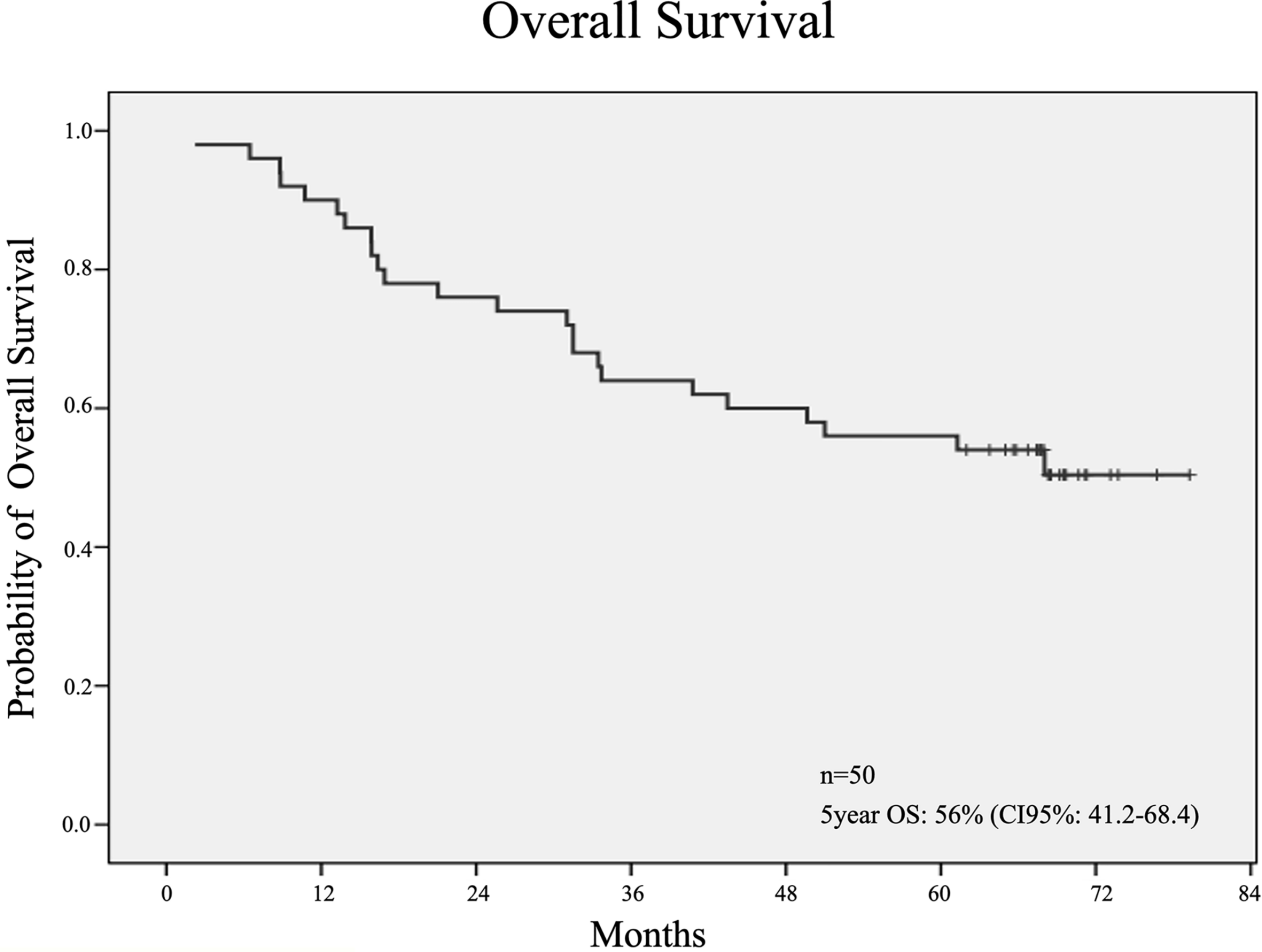
Kaplan Meier curve for OS. n, number; OS, Overall Survival; CI, Confidence Interval.

**Figure 5:**
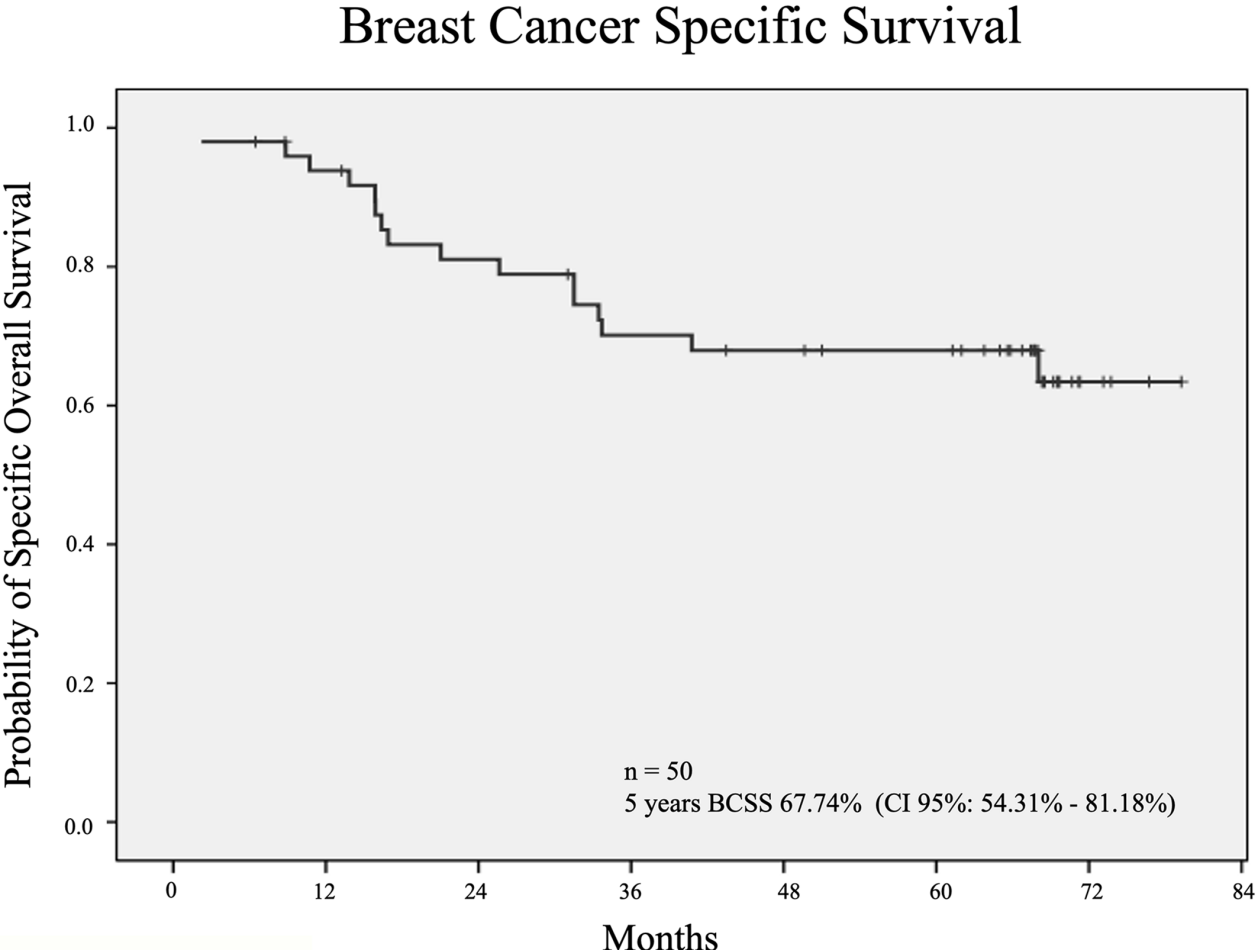
Kaplan Meier curves for BCSS. n, number; BCSS, Breast Cancer Specific Survival; CI, Confidence Interval.

At 60 months of follow-up, median DFS was 50% (95% CI 40.2-68.1), median OS was 56% [IC95%: 41.2-68.4] (**Figure 4**) and breast cancer-specific survival (BCSS) was 67.74% (CI 95%: 54.31% - 81.18%) (**Figure 5**) in the ITT patients. For those patients who underwent surgery, 5 years RFS was 54.4%. [95% CI: 38.3-67.9].

## Discussion

In this prospective phase II trial, PLD 35mg/m^2^ plus cyclophosphamide 600mg/m^2^ every 4 weeks followed by paclitaxel 80mg/m^2^ as NAC was an effective and cardiac safe regimen in a population of breast cancer patients for whom DOX was contraindicated. To our knowledge, this is the first study analyzing cardiac safety, DFS, RFS, OS and BCSS with PLD as NAC for breast cancer at long-term follow-up. Our study demonstrates that this scheme has low risk for cardiotoxicity and is effective in this fragile sub-population, mostly elderly, with very high-risk breast cancer as 88% were triple negative tumors and half of them locally advanced.

The optimal NAC scheme is not well established yet, but combinations of anthracyclines and taxanes have shown the highest rates of pCR (10-31%) (16).

The major late adverse effect of anthracyclines is cardiotoxicity. The incidence of CHF secondary to DOX can manifest even some years after the treatment was administered (17). According to the guidelines of the American Society of Clinical Oncology (ASCO), the risk of developing DOX cardiotoxicity increases 1.6 to 6.8 times in patients over 65 years of age (18). One of the reasons the old heart is particularly vulnerable to cardiotoxicity by chemotherapy is probably the age-related loss of cardiomyocytes. In addition, it is noteworthy that pharmacokinetics of anthracyclines appears to be altered in the elderly, resulting in significant increased DOX concentrations, particularly evident in the heart (19). Finally, aging is generally accompanied by the development of comorbidities, such as hypertension, high blood levels of cholesterol and diabetes mellitus. Thus, those additional risk factors for cardiotoxicity act as a “snowball effect” in elderly patients with cancer, as described by Reddy et al. (20). For all these reasons, the elderly patients or those that have a prior history of heart disease do not usually receive anthracyclines in clinical practise. Furthermore, one of the main caveats of clinical trials in oncology is the limited information available regarding the elderly. Typically, clinical trials with DOX, including PLD, are limited to women up to 70 years old and in optimal performance status (21).

The encapsulation of DOX in liposomes alters its pharmacokinetic properties and is associated with less cardiotoxicity (8–13). There is no standardized test for the assessment of anthracycline-induced cardiotoxicity. The most widespread clinical practice is to conduct a LVEF baseline study and repeat the same exploration periodically during treatment, but long-term LVEF assessment is not common. The role of cardiac damage plasmatic markers is not completely established.

Combination of PLD plus cyclophosphamide has demonstrated little or no cardiotoxicity in three phase II trials in advanced breast cancer patients (22–24). The combination of PLD plus paclitaxel, in the metastatic setting, was also used by Vorobiof et al. who described one asymptomatic LVEF drop > 20% in 26 patients (4%) (25) and Rigatos et al. (26) who reported cardiotoxicity in 2 of the 24 treated patients (8%).

In the neoadjuvant setting, several small studies and two single-arm phase II trials with liposomal anthracyclines have been published showing high response rates, good tolerability and low acute cardiac toxicity (27–29). A recent retrospective study has compared a small cohort of patients treated with PLD regimens such as NAC versus another matched cohort treated with conventional epirubicin-based regimens, showing similar efficacy and less cardiotoxicity evaluated during a 6 month-period (30). Docetaxel 100 mg/m^2^ for 4 cycles, followed by non-pegylated liposomal DOX (Myocet^®^) 60 mg/m^2^ combined with cyclophosphamide 600 mg/m^2^ was analysed in a phase II trial without significant decreases in LVEF during NAC (31). Results of a non-comparative phase II study with the combination of PLD plus cyclophosphamide followed by docetaxel as NAC in breast cancer have recently been published (29). There was no evidence of a significant decrease in mean LVEF values during the 24 weeks of treatment. However, long-term cardiac toxicity and efficacy have not been reported yet in any of those studies.

Our study is the first to report 5-year follow-up of clinical outcome, LVEF monitorization and ECG data. Overall, there was no significant decrease in the median LVEF, no relevant changes in the ECG or late cardiac symptoms. Only 4 patients (8%) presented severe early adverse events from which 2 were ≥G3. In addition, the median age of the patients included in the trial was 73 years old, being 84% over 65. Most of them had high blood pressure (64%) and 10% had history of heart disease. These results support the uphold of the use of PLD in this subset of patients. Age and comorbidities are substantial differences from the rest of the studies published to date; in which the median age ranges between 47 and 54 years old and patients over 70 or with history of heart disease were exclusion criteria (9, 17, 18).

It is noteworthy that although 96% of patients of this trial completed all four cycles of PLD plus cyclophosphamide, but only 52% of patients could complete the 12 weeks planed of paclitaxel mostly due to neurotoxicity, asthenia and HFS. This fact reflects the good tolerance to PLD-cyclophosphamide, but probably poor tolerance to sequentially administration of paclitaxel. Other studies including younger population such as Li et- study have shown that up to 86% of patients were able to complete the treatment with docetaxel but median age was remarkably lower (46 years old) and patients over their 70’s were excluded (28). The hand-foot syndrome (HFS) and skin reactions are typical toxicities associated with PLD (12, 28). A median dose of 10 mg/m^2^ per week has shown to reduce the incidence and severity of mucositis and HFS. We chose the dose of 35mg/m^2^ of PLD every 4 weeks in an attempt to reduce the mucositis and HFS associated with PLD. In our study skin reactions were documented in 32% of patients but only 2 were grade 3 (4%). This toxicity generally began with weekly infusions of paclitaxel. The cutaneous toxicity derived from PLD is a consequence of microtrauma in the blood vessels, which leads to extravasation of the cytostatics into the tissues. This fact may account for the poor skin tolerance to sequentially administered paclitaxel after PLD. Li et al. report 45% of HFS with PLD plus cyclophosphamide followed by docetaxel (29). Most patients in our trial experienced fatigue (84%) but it was ≥ G3 in only 6% of cases. Peripheral sensorial neuropathy, mainly due to paclitaxel was also seen in 62% patients.

Some phase II studies have shown efficacy of PLD as NAC, in combination with cyclophosphamide (22–24, 28) or with paclitaxel (25–27) with ORR ranging from 38% to 80% (**Table S5**). The pCR rate in previously published phase II studies ranges from 8% to 36% (27, 30). The pCR in our study was remarkably high: 32% in the ITT population and 33.3% in triple negative breast cancer patients, as previously published (14). These rates are especially relevant considering that 26 cases were stage III and seven were inflammatory breast carcinoma (28.7% of whom achieved pCR). These results seem superior to the trial by Li et al, where breast pCR rate was 18.75% (95% CI 11.5-26%) using a very similar scheme to us but administering a higher dose of PLD (40 mg/m2) combined with docetaxel instead of paclitaxel (29). This lower pCR rate in their study is probably due to the fact that the population included was much more heterogeneous than that from our study. This is reflected by the fact that the pCR rate amongst the 16 triple negative tumors in that series was 43.75%.

For the 46 patients who underwent surgery, 5-year RFS was 54.4% [95% CI: 38.3-67.9] and 5-year OS for ITT was 56% (IC95%: 41.2-68.4). Long-term OS was probably limited by the elderly population and the significant association with comorbidities. In addition, most of the tumors were triple negative and half of them were locally advanced breast cancers. 5-year BCSS was 67.74% (CI 95%:54.31%- 81.18%). This RFS and BCSS is in line with what has been published in patients with bulky triple negative tumors (32). Furthermore, this therapeutic strategy allowed doubling the BCS rate (26% to 58%) in this very fragile cohort of patients.

Although another alternative would have been to treat patients in our study with carboplatin and/or nab-paclitaxel schedules, these drugs were not the standard of care at the time when this study was planned. Indeed, the addition of neoadjuvant carboplatin to a regimen based on taxanes has shown to significantly increase the proportion of patients achieving a pCR in triple negative breast cancer (33, 34). However, the improvement in OS remains unclear.

One limitation of this study is that it is a single-arm phase II trial and therefore we do not have a direct comparison with a non-anthtracycline chemotherapy regimen. A second limitation of this regimen is the paclitaxel dose-intensity. Only 52% of the patients were able to complete the paclitaxel regimen after PLD plus cyclophosphamide, mainly due to cutaneous and neurological cumulative toxicity. Whether the administration of the drugs in the reverse order would have prevented cutaneous and neurological toxicities and if the administration of paclitaxel would have resulted in an increase of the pCR rate, DFS and OS is unclear and deserves further investigation.

## Conclusions

Our study demonstrated that a PLD-based NAC chemotherapy scheme can lead to equivalent long-term efficacy but with significantly less cardiotoxicity than regimens that include conventional DOX. The good tolerability profile of PLD without compromising effectiveness makes it a favourable choice over conventional anthracyclines in elderly patients or patients with risk factors for cardiac disease. This regimen should be considered as a neoadjuvant treatment option for elderly or cardiotoxicity-prone patients with high-risk breast cancer.

## Data Availability Statement

The raw data supporting the conclusions of this article will be made available by the authors, without undue reservation.

## Ethics Statement

The studies involving human participants were reviewed and approved by Hospital Vall de Hebron Ethics Committee on Clinical Research. The patients/participants provided their written informed consent to participate in this study.

## Author Contributions

MG-G: Conception, study design, data collection, analysis, interpretation of results, figure design, article draft writing. MiB: Data collection, analysis, interpretation of results, figure design. MeB: Drafted the manuscrit. SM, AB, CS, AF, EG-M, NM-J, MM, EC and SP participated in data collection. TP and JC participated in data analysis. XP participated in data analysis and figure design. All authors contributed to the article and approved the submitted version.

## Funding

The rest of the study was supported by the collaborative group SOLTI.

## Conflict of Interest

MG-G declares consulting/advisory fees from Pfizer, Daiichi-Sankyo, Novartis and Roche. MB declares consulting/advisory fees from Pfizer, Novartis and Lilly. SM declares consulting/advisory fees from Pfizer, Roche and Lilly. AB declares consulting/advisory fees from Pfizer, Roche, Bristol Muers Squibb, Astra-Zeneca and Lilly. CS declares consulting/advisory fees from Pfizer, Roche, Macrogenics, Piqur therapeutics, Puma, Synthon and Novartis. EG-M declares consulting/advisory fees from Roche; Astra-Zeneca, Clovis, Pharmamr, Celgene and Novartis. EC declares consulting/advisory fees from Puma, Pfizer, Roche, Astra-Zeneca, Celgene, Daiichi-Sankyo, Eisai, Genomyc Health, Novartis Pierre Fabre and Synthon. SP declares consulting/advisory fees from Astra-Zeneca, Daiichi-Sankyo, Novartis, Polyphor, Roche, and Seattle Genetics; and travel/accommodation grants from Novartis.

The remaining authors declare that the research was conducted in the absence of any commercial or financial relationships that could be construed as a potential conflict of interest.

## Abbreviations

BCS, Breast Conserving Surgery; BCSS, Breast Cancer Specific Survival; CHF, Congestive Heart Failure; DFS, Disease Free Survival; DOX, Doxorubicin; ECG, Electrocardiogram; ER, Estrogen receptor; HFS, Hand-Foot Syndrome; ITT, Intent-to-treat; LVEF, Left Ventricular Ejection Fraction; NAC, Neoadjuvant Chemotherapy; ORR, Overall Response Rate; OS, Overall Survival; pCR, Pathologic complete response; PLD, Pegylated Liposomal Doxorubicin; RFS, Relapse-free survival.
