# Supplementary material for: Long-Term Cardiac Safety and Survival Outcomes of Neoadjuvant Pegylated Liposomal Doxorubicin in Elderly Patients or Prone to Cardiotoxicity and Triple Negative Breast Cancer. Final Results of the Multicentre Phase II CAPRICE Study
Source: Front Oncol. 2021 Jul 9;11:645026. doi: 10.3389/fonc.2021.645026 (PMC8300427; doi:10.3389/fonc.2021.645026)
Supplement: Supplementary file 1 [file DataSheet_1.docx]

**Table S1.** Eligibility Criteria

Inclusion Criteria**:**

- Histologically confirmed invasive breast carcinoma
- Tumour ≥ 2 cm
- ER < 50%
- At least one of the following cardiotoxicity risk conditions: more than 65 years old, arrhythmia requiring treatment (stable at the time of inclusion), valvular disease (stable at the time of inclusion), coronary, left ventricular hypertrophy, history of hypertension requiring pharmacologic treatment lasting ≥ 3 years, LVEF < 55% or prior DOX (≤ 300 mg/m2).
- Patients were required to have adequate bone marrow function defined as WBC > 2500, absolute granulocyte count > 1500 cells/L, platelet count > 100.000 cells /L, and hemoglobin concentration > 8g/dL;
- Adequate liver function, defined as ALT less than 5 the upper limit of normal and a bilirubin concentration < 1.5 mg/dL
- Adequate renal function, defined as a clearance of creatinine > 30 ml/min.:

Exclusion Criteria:

- Severe CHF (NYHA III-IV),
- Myocardial ischemia < 6 months before
- Ductal carcinoma Grade 1 or Colloid or Lobular Carcinoma
- Metastatic disease
- Karnofsky Performance Status < 70%,
- LVEF < 45%
- Knowledge that the tumor overexpressed or amplified HER2

Abbreviations: ER: estrogen receptors, DOX: doxorubicin, LVEF: Left ventricular ejection fraction, WBC: White blood count, CHF: Congestive Heart Failure, ALT: Alanineaminotransferas.
