# Supplementary material for: Long-Term Cardiac Safety and Survival Outcomes of Neoadjuvant Pegylated Liposomal Doxorubicin in Elderly Patients or Prone to Cardiotoxicity and Triple Negative Breast Cancer. Final Results of the Multicentre Phase II CAPRICE Study
Source: Front Oncol. 2021 Jul 9;11:645026. doi: 10.3389/fonc.2021.645026 (PMC8300427; doi:10.3389/fonc.2021.645026)
Supplement: Supplementary file 2 [file DataSheet_2.docx]

**Table S3.** Statistical Definitions

- Patients in intention to treat (ITT) all patients who began treatment with PLD.
- Patients operated: Composed set all ITT patients who have undergone surgical treatment. The main and secondary objectives concerning radiological response, safety and tolerability were assessed in the ITT set. For this, in cases where it lacks the necessary information it was assumed the worst case scenario.
- Patient survival and progression-free intervals were measured from time of entry into the protocol.
- Toxicity was assessed through the use of descriptive statistics.
- Secondary objectives concerning the frequency of conservative surgery and the frequency of patients with axillary node involvement was assessed in all patients operated.
- The analysis for the adverse events and cardiac safety have been the description of the frequencies that have been presented different degrees of cardiotoxicity as classified by LVEF (CTC AE version 3), and in the calculation of the proportion of patients who have presented a grade 2 or greater accompanied by confidence intervals (bilateral) of 95%.The analysis of adverse events (general and cardiac) include descriptions of the frequencies of patients who have had these adverse events, classifying them in advance by the appropriate terms in the dictionary CTC AE. Frequencies have been presented short terms (AE short name) and categories (category) anatomophysiological.
- The analysis of relapse-free survival and overall survival at 5 years after surgery will be performed by estimating the survival function by the Kaplan-Meier method, and the estimated median survival with her range confidence.
