# Supplementary material for: Long-Term Cardiac Safety and Survival Outcomes of Neoadjuvant Pegylated Liposomal Doxorubicin in Elderly Patients or Prone to Cardiotoxicity and Triple Negative Breast Cancer. Final Results of the Multicentre Phase II CAPRICE Study
Source: Front Oncol. 2021 Jul 9;11:645026. doi: 10.3389/fonc.2021.645026 (PMC8300427; doi:10.3389/fonc.2021.645026)
Supplement: Supplementary file 3 [file Table_1.docx]

**Table S2:** Dose reduction criteria

| **Cardiac toxicity criteria** | | |
| --- | --- | --- |
| CHF severe | Withdrawal | |
|  |  |  |
| CHF moderately symptomatic or  Asymptomatic | LVEF < 45%: withdrawal | |
|  | LVEF decrease 10-20 points and LVEF ≥45%: keep treatment | |
|  | LVEF decrease > 20 points : withdrawal | |
| **Hematology toxicity criteria** | | |
| **PLD+CPM** | | |
| 0.5x10^9^ /l ≥ *N* < 1.5x10^9^ /l and/or  25x10^9^/l ≥ Plat < 75x10^9^/l | interrupted until *N* < 1.5x109 /l and  Plat < 75x109/l | No dose reduction |
| *N* < 0.5x109 /l and/or Plat < 75x10^9^/l | Interrupted until *N* < 1.5x109 /l and  Plat < 75x109/l | Dose reduction of 15% for both drugs |
| If a patient needs more than 2 weeks to recover normal levels of neutrophils or platelets, PLD plus CO was   withdrawal and patient start PYX treatment or surgery was performed at investigator criteria | | |

| **Paclitaxel** | | | | | | | | | | | |
| --- | --- | --- | --- | --- | --- | --- | --- | --- | --- | --- | --- |
| *N* < 1.0x109 /l and/or Plat < 75x109/l | | | Interrupted until *N* < 1.5x109 /l and  Plat < 75x109/l | | | | Recovery ≤ 1 week: no dose reduction | | | | |
|  |  |  |  |  |  |  | Recovery ≤ 2 week: rechallenge at a   20 % dose reduction | | | | |
| If a patient needs more than 2 weeks to recover normal levels , paclitaxel was withdrawal and surgery was performed | | | | | | | | | | | |
| **Non-hematology toxicity criteria** | | | | | | | | | | | |
| **PLD+CPM** | | | | | | **PTX** | | | | | |
| Hand foot syndrome (HFS) | | | | | | Adverse Events | | | | | |
| G1 | Treatment not  interrupted | | | No dose reduction | | G1 | | Treatment not   interrupted | | | |
| G2 | interrupted until  ≤ G1 | | | No dose reduction | | G2 | | Treatment not  interrupted | | | |
| G3 | interrupted until  ≤ G1 | | | 15% dose reduction | | G3 | | interrupted until   ≤ G1 | | | |
| G4 | interrupted until  ≤ G1 | | | 25% dose reduction | | G4 | | interrupted until  ≤ G1 | | | |
| If a patient needs more than 2 weeks to G1, withdrawal PLD+CP  treatment and start with PTX | | | | | | If a patient more than 2 weeks to recover to G2, withdrawal PTX  treatment and surgery performed | | | | | |
| Mucositis | | | | | | Hypersensitivity | | | | | |
| G1 | | Treatment not  interrupted | | | No dose reduction | Immediately discontinued the infusion and appropriate symptomatic treatment. Treatment was restarted a lower speed. If tolerated, the infusion may then be completed over the next hour to a total of 90' | | | | | |
| G2 | | interrupted until  ≤ G1 | | | No dose reduction |  |  |  |  |  |  |
| G3 | | interrupted until≤ G1 | | | 15% dose reduction | **PLD+CPM, paclitaxel** | | | | | |
|  |  |  |  |  |  | Bilirubin > 2x normal lab value | | | | | |
| G4 | | interrupted until≤ G1 | | | 25% dose reduction | Treatment interrupted | | | | recovery in 3  weeks to G1 | Treatment not  interrupted |
| If patient needs more than 2 weeks to G1, withdrawal PLD+CP  treatment and start with PTX | | | | | |  |  |  |  | no recovery in 3 weeks  to G1 | Interrupted treatment  and surgery performed |
| Anaphylactic reaction during infusion | | | | | | Creatinine clearance  40-156 ml/min | | | Normal dose | | |
| Immediately discontinued the infusion and appropriate  symptomatic treatment. Treatment was restarted at a lower  speed. If tolerated, the infusion may than be completed over  the next hour to a total of 90 ' | | | | | |  |  |  |  |  |  |
|  |  |  |  |  |  | Creatinine clearance  ≤ 40 ml/min | | | If ≤ 3 weeks to normal, no interrupted treatment | | |
|  |  |  |  |  |  |  |  |  | If ≥3 weeks to normal, interrupted treatment and surgery performed | | |

Abbreviations: CHF: Congestive Heart Failure, LVEF: Left ventricular ejection fraction, N: Neutrophils, Plat: Platelels, G: Grade, PLD: Pegylated liposomal doxorubicin, CPM: Cyclophosphamide, PTX: Paclitaxel,
