# Supplementary material for: Long-Term Cardiac Safety and Survival Outcomes of Neoadjuvant Pegylated Liposomal Doxorubicin in Elderly Patients or Prone to Cardiotoxicity and Triple Negative Breast Cancer. Final Results of the Multicentre Phase II CAPRICE Study
Source: Front Oncol. 2021 Jul 9;11:645026. doi: 10.3389/fonc.2021.645026 (PMC8300427; doi:10.3389/fonc.2021.645026)
Supplement: Supplementary file 4 [file Table_2.docx]

**Table S4.** Pathological Responses

|  | **pRC in breast**  **n (%)**  [95% CI] | **pCR breast & axilla**  **n (%)**  [95% CI] |
| --- | --- | --- |
| Total | 50 (100%) | 50 (100) |
| **Yes** | **16 (36)**  [19.5-46.7%] | **12 (24)**  [12.1-35.8%] |
| No | 34 (64) | 38 (76) |

Abbreviations: pCR: pathological complete response, CI: confidence interval.
