# Supplementary material for: Long-Term Cardiac Safety and Survival Outcomes of Neoadjuvant Pegylated Liposomal Doxorubicin in Elderly Patients or Prone to Cardiotoxicity and Triple Negative Breast Cancer. Final Results of the Multicentre Phase II CAPRICE Study
Source: Front Oncol. 2021 Jul 9;11:645026. doi: 10.3389/fonc.2021.645026 (PMC8300427; doi:10.3389/fonc.2021.645026)
Supplement: Supplementary file 5 [file Table_3.docx]

**Table S5.** Cardiac toxicity and clinical efficacy associated to liposomal doxorubicin in breast cancer trials

| **STUDY**  *Author (Ref)*  *Design* | **N** | **Setting** | **Antracycline** | **Schedule** | **HER2** | **Median age** | **Cardiac assessment** | **Cardiotoxicity** | **Efficacy** |
| --- | --- | --- | --- | --- | --- | --- | --- | --- | --- |
| *O’Brien (12)*  *Phase III* | 509 | 1^st^ line MBC | PLD vs DOX | Monotherapy | No | 58.5 | LEVF at baseline, one during treatment after 300 and 400mg/m2 LVEF | **DOX > PLD** (HR = 3.16; 95%CI 1.58–6.31; *P* <0.001) | PFS 6.9 vs 7.8 m HR 1 (95%CI 0.82-1.22)  OS 21 vs 22m HR 0.94; (95%CI 0.74–1.19) |
| *Rafiyath (13)*  Metanalysis | 2220 | 1^st^ line MBC | Liposomal vs conventional antracyclines | Monotherapy | No | Patients with median ages between 37 and 59 | Congestive cardiac failure and mean percentage change in LVEF from baseline | **Conventional > Liposomal** OR 0.34 (95%CI 0.24-0.47) | Not evaluated |
| *Overmoyer (22)*  *Phase II* | 51 | 1^st^ line MBC | PLD 30mg/m2/3w | Plus CPM 600mg/m2 | No | 54 | LEVF at baseline, accumulative dose of 300mg/m2, each 100 mg/m2 thereafter and at the end. | **LVEF decrease** (G1) in 15%  All asymptomatic | ORR 50%, CR 8%, PR: 43%, CB: 86%. |
| *Trudeau (23)*  *Phase II* | 70 | 1^st^ line MBC | PLD 35 mg/m2 | Plus CPM 600mg/m2/3w | No | 55 | LEVF at baseline every 2 cycles | **1.4% pts had > 15% in LVEF** drop  7.14% pts had > 10% LVEF drop at the end of treatment.  All asymptomatic | ORR 38%. BC: 71% PD: 29% TTP: 12.2 m OS: 16.5 m. |
| *Rau (24)*  *Phase II* | 45 | 2^nd^ line MBC | PLD 40mg/m2/3w | Plus CPM 500mg/m2 5FU 500mg/m2/3w | No | 52.5 | LVEF at baseline at the end of treatment | **No decrease in LVEF** | ORR: 80%; PD 15.6% PFS 8.2m OS 36.6m |
| *Vorobiof (25)*  *Phase II* | 34 | 1^st^ line MBC | PLD 30mg/m2/3w | Plus paclitaxel 175mg/m2 | No | 55 | LEVF at baseline and at the end. | LVEF decrease > 20% (**G2**) in 3%  LVEF decrease >10% (**G1**) in 20%.  All asymptomatic | ORR 73%, CR 21% PR 53% PD 3%. |
| *Rigatos (26)*  *Phase II* | 23 | 1^st^ line MBC | PLD 30mg/m2/3w | Plus paclitaxel 175mg/m2 | No | 59 | LEVF at baseline, and at the end of treatment | Significant drop in LVEF in one pts and one arrhythmia (8.7%). All asymptomatic | ORR: 69.57%. CR 8.70% PR 60.87%. TTP: 7 m, OS: 10 m. |
| *Dong (30) Phase II matched 1:2* | 43/86 | **NAC** | PLD 35mg/m2/3w vs epirrubicin 100mg/m2/3w | Plus taxanes | No | 51 | LEVF was measured at baseline, and during treatment | **Non-significant differences in LVEF drop rate>10%** (p=0.463) | ORR PLD 76.6% epirrubicin 75.7%  PD both 2.3%  **pCR: 16.3%** vs 11.6% |
| *Gogas (28)*  *Phase II* | 35 | **NAC** | PLD 35 mg/m2/3w | Plus paclitaxel 175mg/m2 | No | 54 | LEVF was measured at baseline and during treatment | **No significant changes during treatment.** | ORR 71% CR 17%, PR: 54% PD 6% **pCR:8.5%** |
| *Schmid (27)*  *Phase II* | 44 | **NAC** | Non peylated liposomal DOX 60mg/m2/3w | Plus docetaxel 75mg/m2 and gemcitabine 350mg/m2 /3w | No | 45 | LEVF at baseline and every 2 cycles. Serial ECG | **No cases of cardiac failure** | ORR: 73%. CR: 23% PR 50% PD: 2.5% **pCR: 16%** |
| *García Mata (31)*  *Phase II* | 74 | **NAC** | Non-pegylated liposomal DOX 60m/m2/3w | Docetaxel 75mg/m2 and CPM 600mg/m2 | No | 46 | LEVF at baseline, and during treatment | **No significant changes in LVEF** | ORR: 75**%,** PD: 2% **pCR 24%** |
| *Gil-Gil (14)* | 50 | **NAC** | PLD 35 mg/m2/4w | Plus CPM 600mg/m2/4w followed paclitaxel 80 /w | No | 73 | LEVF at baseline, 9, 6 and 18 w and **during 5 years** | **No significant changes in LVEF** | ORR 26%; **pCR 32%,** **5y RFS 54.4%, 5y OS 56%and 5y BCSS 67,7%** |

Abbreviations: CB, Clinical Benefit; CI, Confidence Interval; CPM, Cyclophosphamide; CR, Complete Response; DOX, Doxorubicin; m, months; ECG, Electrocardiogram; G, Grade; HR, Hazard Ratio; LVEF, Left ventricular ejection fraction; N, number; NAC; Neoadjuvan chemotherapy, MBC, Metastatic Breast Cancer, ORR, Overall Response Rate; OS, Overall Survival; pCR, Pathological Complete Response; PD, Progression; PFS, Progression Free Survival; PLD, Pegylated liposomal doxorubicin; Pts, Patients; PR, Partial Response; Ref, Reference,TTP, Median time to progression; w, weeks; y, year
